# Effect of Patient Participation on Nurse and Patient Outcomes in Inpatient Healthcare

**DOI:** 10.3390/ijerph16081344

**Published:** 2019-04-15

**Authors:** Bin Ding, Wei Liu, Sang-Bing Tsai, Dongxiao Gu, Fang Bian, Xuefeng Shao

**Affiliations:** 1 Zhongshan Institute, University of Electronic Science and Technology of China, Guangdong 528400, China; bianfangzsc@hotmail.com; 2 Department of Management & Organization, Robert H. Smith School of Business, University of Maryland, College Park, MD 20742, USA; 3 Discipline of International Business, The University of Sydney, Sydney 2006, Australia; wei.liu2@sydney.edu.au; 4 The School of Management, Hefei University of Technology, Hefei 230009, China; gudongxiao@hfut.edu.cn; 5 La Trobe University Sydney Campus, Sydney 2000, Australia; x.shao@latrobe.edu.au

**Keywords:** patient participation, patient satisfaction, nurse-patient relationship, job satisfaction, work engagement, helping behaviors

## Abstract

Using service-dominant logic as a theoretical lens, this study investigated the co-production of healthcare service and service value co-creation between nurses and patients. The main objective of this study was to: (1) examine the effect of patient participation on patient satisfaction and nurses’ attitudes and behaviors; (2) examine boundary conditions of the effect of patient participation on patients and nurses. We proposed that patient participation positively impacted patient satisfaction and nurse job satisfaction, work engagement, and helping behaviors. We further proposed that first inpatient stay and length of stay moderated the effect of patient participation on patient satisfaction, and nurses’ sociodemographic characteristics moderated the effect of patient participation on nurse job satisfaction, work engagement, and helping behaviors. Using survey data from 282 nurses and 522 inpatients from a public hospital in China, we found that the effect of patient participation on patient satisfaction was contingent upon first inpatient stay and length of stay. We also found that patient participation improved nurse job satisfaction, work engagement, and helping behaviors. Furthermore, nurses’ sociodemographic characteristics, namely age and organizational tenure, moderated the effect of patient participation on nurse job satisfaction, but not on work engagement and helping behaviors. Theoretical and practical implications of our findings were discussed.

## 1. Introduction

Patient participation in healthcare has received increasing attention in both practice and academia. The Department of Health has announced that involving patients and citizens in healthcare service is a central theme of national and local policy in the National Health Service [1]. Patient participation was one of the World Health Organization’s (WHO) calls to action in 2013. The WHO encourages national health systems to engage participants in hand hygiene and patient safety improvements, and the organization has developed guidelines and programs to assist with implementation [2]. China is undergoing a reform process that transitions its health care delivery system toward a people-centered, high quality, and integrated one [3]. A people-centered integrated care service delivery system is proposed, aiming at improving healthcare services, enhancing quality of care and reducing costs [3,4,5]. One of the key strategic directions of this system is to engage patients to make better decisions about their health. In academia, researchers have demonstrated that patient participation plays a non-negligible role in enhancing healthcare service, including the nurse–patient relationship, shared decision making, chronic disease treatment, and a redesign of healthcare systems [6,7,8,9,10,11].

Involving patients in healthcare is universally rewarding for patients, communities, healthcare professionals, and healthcare delivery systems [1]. Past research has widely shown the positive influence of patient participation on patients, most notably on patient satisfaction, patient safety, quality of healthcare, patient psychological well-being, and clinical outcomes [7,12,13,14,15]. Research on the impact of patient participation on healthcare professionals has been much less. The Department of Health points out that involving patients in treatment and healthcare service delivery can bring various benefits to healthcare professionals, for example, enhancing their understanding of patients’ health problems, better management of the consultation process, helping them gain patients’ trust, and enabling them to deliver desired healthcare services [1]. Similarly, in a policy report, the Chinese government together with the World Bank Group and the WHO, proposes that a people-centered healthcare system with engaged patients may benefit health professionals and result in improved job satisfaction, reduced burnout and role enhancement [3]. Although the potential benefits of involving patients on healthcare professionals are intriguing, it has not been subject to rigorous empirical examination, especially with a Chinese sample. Against this backdrop, this study sought to examine how patient participation benefits healthcare professionals, above and beyond its impact on patients with a Chinese sample.

Using the service-dominant logic framework in service marketing research as a theoretical basis, the current study delineates a co-production dual process model of healthcare service between nurses and patients [14,16]. We consider nurses as the main healthcare service providers in this study for the following reasons. First, we are interested in studying inpatient healthcare service delivery, in this context, nurses are the main health professionals that interact with patients. Second, nurses engage in frequent interactions with patients during inpatient care, predominantly on a daily basis, and hence providing patients with ample opportunities to participate in a service co-production process. Third, a large body of research on patient participation considers nurses as the service providers and patients as the service recipients, for example, [6,17,18,19]. 

In our co-production dual process model, we propose that patient participation in healthcare makes nurses and patients co-producers of healthcare service, and the value of this co-production benefits both nurses and patients. The objective of this research is threefold. First, beyond examining the direct effect of patient participation on patient satisfaction, we investigate patient participation from the nurses’ perspective and how it impacts nurses’ attitudes and behaviors, including job satisfaction, work engagement, and helping behaviors. Second, because extant empirical research has shown that patient participation does not necessarily lead to increased patient satisfaction or improved treatment outcomes, we examine hospital stay factors, such as first time of inpatient stay and length of inpatient stay, as boundary conditions of the effect of patient participation [20,21,22]. Investigating hospital stay factors is important because they are alterable to allow service providers, managers, and policy makers to intervene and make improvements [23]. Third, we argue that the effect of patient participation on nurses’ attitudes and behaviors may be contingent upon nurses’ sociodemographic characteristics, such as age, sex, education, and organizational tenure. Therefore, we are interested in examining nurses’ sociodemographic characteristics as boundary conditions for the effect of patient participation on nurses’ job satisfaction, work engagement, and helping behaviors. The rest of this paper is organized as follows: we first elaborate on the theoretical underpinning of co-production and value co-creation in healthcare services, and introduce a series of theoretical hypotheses. We then present the method and results of the current research. Finally, we discuss the theoretical and practical implications of our findings.

## 2. Theory and Hypothesis Development

### Patient Participation, Co-Production, and Value Co-Creation in Healthcare

Brownlea defined participation as “getting involved or being allowed to become involved in a decision-making process or the delivery of a service or the evaluation of a service, or even simply to become one of a number of people consulted on an issue or a matter” ([24], p. 605). Along this line of thought, we view patient participation as the collaboration between nurses and patients in various forms during the process of healthcare service delivery. Following Chan et al., we define patient participation as a behavioral construct that measures the extent to which patients provide/share information, make suggestions, and become involved in healthcare [25]. In the service marketing literature, Vargo and Lusch proposed the service-dominant logic (SDL) framework, which emphasizes the crucial roles of both service providers and customers [16]. Besides being recipients of service, customers are viewed as co-creators of service, according to SDL. Co-production between service providers and customers happen through customer participation in the service delivery process [16,26]. In the healthcare setting, previously, the relationship between the patients and nurses follows a “paternalist” model where nurses offered the service and patients played minimal and passive roles [7]. Involving patients in healthcare makes the patients’ role different than it once was [27,28]. Patients no longer passively receive healthcare service; instead, they provide information for diagnosis, express concerns, ask questions, describe symptoms, participate in decision making together with nurses or doctors, and evaluate the service they receive [14]. These behaviors trigger service co-production between patients and nurses wherein both nurses and patients are actively involved in the process. As a result, both are potentially affected by the co-production. For patients, the value of co-production is manifested in improved treatment management, better health outcomes, increased patient safety, improved satisfaction, and so forth [7,20]. For nurses, patient participation offers valuable input for diagnosis and treatment, helps nurses adjust their behaviors to improve care quality, and potentially helps improve the healthcare system. In this way, the co-production between patients and nurses brings benefits for nurses above and beyond those for patients. To gain a clearer and deeper understanding of the influence process of patient participation, we investigate patient participation from the perspectives of both patients and nurses. We simultaneously examine the effect of patient participation on patient satisfaction and nurses’ job satisfaction, work engagement, and helping behaviors towards patients. Numerous studies have considered prior hospitalization and length of stay as important factors in inpatient healthcare [23,29,30]. For example, Thi and colleagues find that patients with longer stay are less satisfied and are less likely to recommend the hospital to others [23]. Soufi and colleagues find that patients with more than two prior hospitalization are more satisfied than those with no prior hospitalization or less than two prior hospitalization [30]. Following prior research, we consider first time of inpatient stay and length of inpatient stay as boundary conditions of the effect of patient participation on patient satisfaction. We focus on hospitalization factors because they can provide important information for managers and policy makers to make effective interventions accordingly. Furthermore, besides examining the direct effect of patient satisfaction on nurse job satisfaction, work engagement, and helping behaviors, we contend that the benefits of patient satisfaction on nurses may vary according to nurses’ sociodemographic characteristics, such as age, sex, education level, and organizational tenure. We will thus examine these sociodemographic factors as boundary conditions for the effect of patient participation on nurses. Taking into account the above arguments, we present our hypotheses below:

**Hypothesis** **1.** *Patient participation is positively related to patient satisfaction.*

**Hypothesis** **2.** *(a) First inpatient stay and (b) length of stay moderate the effect of patient participation on patient satisfaction.*

**Hypothesis** **3.** *Patient participation is positively related to nurse (a) job satisfaction, (b) work engagement, and (c) helping behaviors.*

**Hypothesis** **4.** *Nurses’ sociodemographic characteristics, including age, sex, education, and organizational tenure moderate the effect of patient participation on nurse (a) job satisfaction, (b) work engagement, and (c) helping behaviors.*

## 3. Method

### 3.1. Sample

We collected data from a public hospital in north China using a survey. Public hospitals accounted for almost half of the total number of hospitals in China. Public hospitals were the first choice for most people for seeing a doctor and receiving hospitalization and medical treatment. The public hospital in this study was a general hospital including various departments, such as respiratory, cardiology, otolaryngology, etc. Each department had their wards for inpatient care where nurses and patients had frequent interactions. We first obtained permission from the nursing department head before we started collecting data from nurses. The research protocol was approved by the University of Lausanne (Project No.: #71127-3660210). All nurses at the facility were invited to participate in this study. The researcher distributed paper-and-pencil questionnaires to the nurses, and requested the participants to complete the questionnaires during work shifts onsite. The nurses were requested to return the questionnaires in sealed envelopes after completion, and completed questionnaires should be returned to the nurse stations. After distributing the questionnaires, the researcher emphasized to the participants that they should complete the questionnaires on their own and should not discuss their responses with their colleagues. One week after the questionnaires were distributed, we received 292 completed questionnaires. The total number of nurses in the hospital was 400, and hence the response rate was 73%. The mean age of the nurse respondents was 29.18 years (standard deviation = 8.34). Female nurses accounted for 97.6% of all the participants. The average organizational tenure was 82.61 months (standard deviation = 78.95). More than 41.09% of the respondents had an education level higher than college. 

In our sample, we noted that a small portion of the nurse participants had education level below high school (*n* = 10), which we considered to be invalid responses, because normally nurses were required to obtain high school degree. Due to this reason, we deleted nurses whose education was lower than high school for hypothesis testing. The final sample size was 282. Table 1 presents the characteristics of the nurse sample.

We collected patient data two weeks after we collected the nurse data. Because we wanted to target on inpatients, we walked into inpatient wards to invited patients to participate onsite. After patients expressed their willingness to join in our survey, we confirmed their inpatient status again. Due to low patient literacy levels, most of the time, the researchers read the questionnaire questions to the participants and circled the answers on their behalf. Patient data collection lasted for one week. A total of 522 patients participated in the survey. The mean age of the patient respondents was 52.39 years (standard deviation = 16.89). Females accounted for 53.45% of all patient participants, and 68.95% of the patients had prior hospitalization experience. The average length of current inpatient stay was 9.29 days (standard deviation = 8.41). Table 2 presents the characteristics of the patient sample. 

### 3.2. Measure Operationalization

We adopted existing measures for all the variables in this study and slightly adjusted the items to reflect the hospital context. The original questionnaire was prepared in English and then translated into Chinese using standard back translation for distribution in China [31].

#### 3.2.1. Patient Questionnaire

*Patient participation.* Patient participation was measured with a five-item scale developed by Chan, Yim, and Lam (2010) [25]. A sample item included, “I put a lot of effort into expressing my personal needs to the nurses during the health care service process.” Patients rated patient participation on a 5-point Likert scale ranging from 1, “strongly disagree” to 5, “strongly agree”.

*Patient satisfaction.* Patient satisfaction was measured with a two-item scale, including “Overall, I am satisfied with the service provided by this hospital” and “I am satisfied with my experience in this hospital”. Patients rated patient satisfaction on a 5-point Likert scale ranging from 1, “strongly disagree” to 5, “strongly agree”. 

*First inpatient stay.* This variable was measured by asking patients one question: “Is this your first inpatient stay?” Patients answered by choosing either “yes” or “no”. 

*Length of stay.* This variable was measured with one item: “How long will you stay in the hospital this time?” Patients answered by choosing from among “Less than three days”, “three to seven days”, “one to two weeks”, “two to three weeks”, “three weeks to one month”, “more than one month”. 

*Control variables.* We controlled for patients’ age, sex, and education. Sex was measured with a dummy variable, where 1 represented male and 0 represented female. Patients chose their education level from among “Below high school”, “High school”, “Technical degree”, “Bachelor” and “Master or above”. We also included patients’ contact frequency with nurses as a control variable. Contact frequency was measured by asking patients, “On average, how many times do you interact with the nurses per day?” Patients chose their answers from among “less than 2 times”, “3–5 times”, “6–10 times” and “more than 10 times”. 

#### 3.2.2. Nurse Questionnaire

*Patient participation.* Patient participation was measured with the same scale used in the patient questionnaire, but with wording adjusted for nurses. A sample item included, “Patients put a lot of effort into expressing their personal needs to me during the healthcare service process”. Nurses rated patient participation on a 5-point Likert scale ranging from 1, “strongly disagree” to 5, “strongly agree”.

*Job satisfaction.* Job satisfaction was measured with a four-item scale that measured nurses’ satisfaction with their promotion, salary, bonus, and overall satisfaction. Sample items included “Overall, are you satisfied with your current job?” and “Are you satisfied with the opportunities for promotion in this hospital?” Nurses rated job satisfaction on a 5-point Likert scale ranging from 1, “to a small extent” to 5, “to a great extent”.

*Work engagement.* Work engagement was measured with the Utrecht Work Engagement Scale (UWES) developed by Schaufeli et al. [32]. Sample items included “When I get up in the morning, I feel like going to work”, “I am enthusiastic about my work” and “Time flies when I am working”. Respondents rated their work engagement on a 5-point Likert scale ranging from 1, “never” to 5, “always”. 

*Helping behaviors.* Helping behaviors were measured with a 5-item scale used in Schneider et al. [33]. A sample item included, “I frequently go out of my way to help a patient”. Nurses rated helping behaviors on a 5-point Likert scale ranging from 1, “strongly disagree” to 5, “strongly agree”.

*Sociodemographic characteristics.* We used age, sex, education, and organizational tenure to measure nurse’ sociodemographic characteristics. Sex was measured with a dummy variable whereas 1 represented male and 0 represented female. Nurses chose their education level among “Below high school”, “High school”, “Technical degree”, “Bachelor”, “Master or above”. Organizational tenure was measured by asking “How long have you worked in this hospital?” Organizational tenure was measured in months. 

*Control variables.* We included the average number of patients a nurse contacted per day as the control variable, because this variable reflected the nurses’ workload and might impact nurses’ job satisfaction, work engagement, and helping behaviors. This construct was measured using the question, “On average, how many patients do you interact with every day?” 

## 4. Results

### 4.1. Descriptive Statistics

Table 3 presents the means, standard deviations, and the correlations among the variables. Internal consistency reliabilities were examined with Cronbach’s alpha, which are shown on the diagonal. As shown in Table 3, all the measures had Cronbach’s alpha equaling or greater than 0.70, the threshold for acceptable internal consistency [34]. 

### 4.2. Measurement Model

We conducted a confirmatory factor analysis (CFA) to assess the measurement model for patients and nurses separately. CFA was performed with Stata 14.0 (StataCorp, College Station, TX, USA) with maximum likelihood as the estimation method. For patients, we specified two factors, namely patient participation and patient satisfaction. We constrained the items to load on their corresponding factors, and the two factors were allowed to correlate with each other. Model fit indices indicated that our model fitted the data well: *χ*^2^ (9) = 27.59, *p* < 0.05; Root Mean Square Error of Approximation (RMSEA) = 0.06; Confirmatory Fit Index (CFI) = 0.99; Tucker–Lewis Index (TLI) = 0.99; Standardized Root Mean Square Residual (SRMR) = 0.03. All factor loadings were significant.

For nurses, we specified four factors, namely patient participation, job satisfaction, work engagement, and helping behaviors. We constrained the items to load on their corresponding factors, and the four factors were allowed to correlate with one another. We had satisfactory model fit indices: *χ*^2^ (551) = 1666.60, *p* < 0.001; RMSEA = 0.09; CFI = 0.82; TLI = 0.80; SRMR = 0.08. All factor loadings were significant. The high factor loadings and the good model fit statistics for both patient data and nurse data indicated satisfactory validity of our measures.

### 4.3. Hypothesis Testing

We tested our hypotheses using hierarchical multiple regressions with Stata 14.0. Hierarchical multiple regression allowed us to determine the variance independent variables and interaction terms contributed to the outcomes variables, respectively. We entered the independent variables and interaction terms in separate steps. Hypothesis 1 proposed that patient participation was positively related to patient satisfaction. Model 1 in Table 4 shows that patient participation was not significantly related to patient satisfaction (*b* = 0.09, *p* > 0.05). Therefore, Hypothesis 1 was not supported. 

Hypothesis 2 proposed that first inpatient stay and length of stay moderated the effect of patient participation on patient satisfaction. As shown in Model 2 in Table 4, there was a marginal significant interaction between patient participation and first inpatient stay (*b* = −0.70, *p* < 0.10). To examine the simple effect, we tested the effect of patient participation on patient satisfaction separately for those who stayed for the first time (first inpatient stay = 1) and not for the first time (first inpatient stay = 0). 

**Figure 1:**
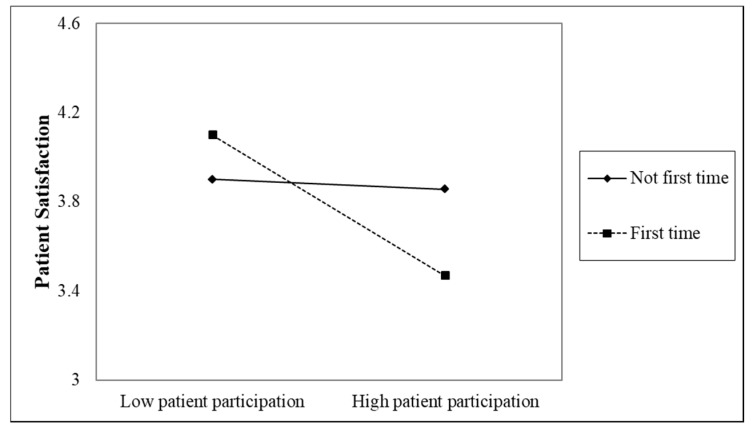
Simple slopes for the interaction between patient participation and first inpatient stay on patient satisfaction.

As shown in Figure 1, for first-time inpatients, increasing patient participation resulted in decreased patient satisfaction (*r* = −0.75, *p* < 0.10); for non-first-time inpatients, increasing patient participation did not significantly affect patient satisfaction (*r* = −0.05, *p* > 0.05). Table 4 also shows that there was significant interaction between patient participation and length of stay (*b* = 0.05, *p* < 0.05). To examine the simple effect, we used one standard deviation above and below the mean to denote high and low levels of length of stay and patient participation. 

**Figure 2:**
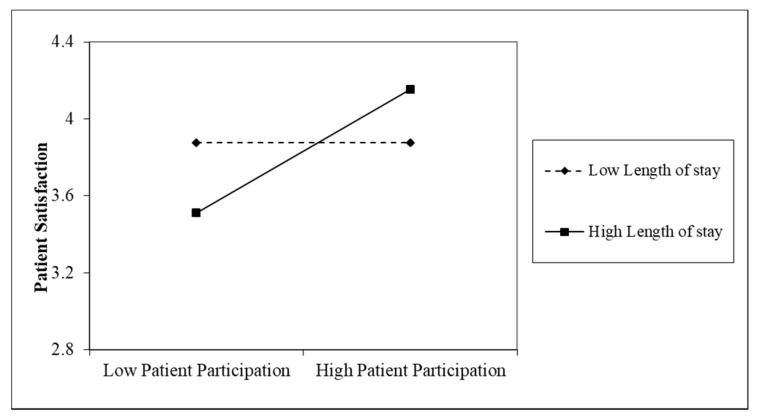
Simple slopes for the interaction between patient participation and length of stay on patient satisfaction.

Figure 2 shows that for those who stayed for a longer period, patient participation was significantly positively related to patient satisfaction (*r* = 0.82, *p* < 0.01); for those who stayed for a shorter period, patient participation was not significantly related to patient satisfaction (*r* = −0.00, *p* > 0.05). Therefore, Hypothesis 2a and Hypothesis 2b were supported. 

Hypothesis 3 proposed that patient participation was positively related to nurse job satisfaction, work engagement, and helping behaviors. Results are shown in Table 5, Table 6 and Table 7. Model 1 in Table 5 shows that patient participation was significantly related to job satisfaction (*b* = 0.25, *p* < 0.001). Model 1 in Table 6 shows that patient participation was significantly related to work engagement (*b* = 0.30, *p* < 0.001). Model 1 in Table 7 shows that patient participation was significantly related to helping behaviors (*b* = 0.22, *p* < 0.001). Therefore, Hypothesis 3 was supported. 

**Figure 3:**
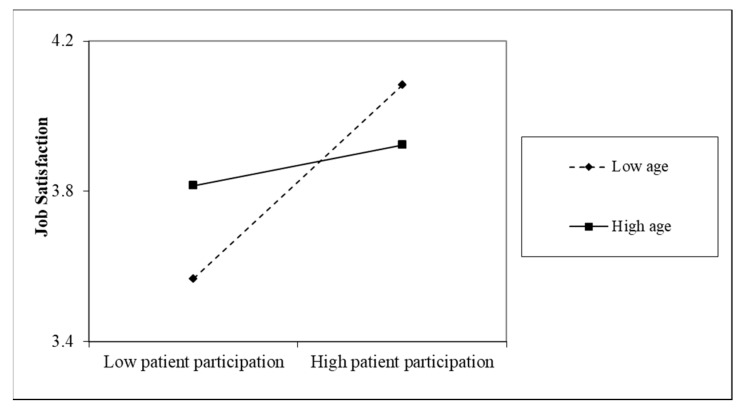
Simple slopes for the interaction between patient participation and age on nurse job satisfaction.

**Figure 4:**
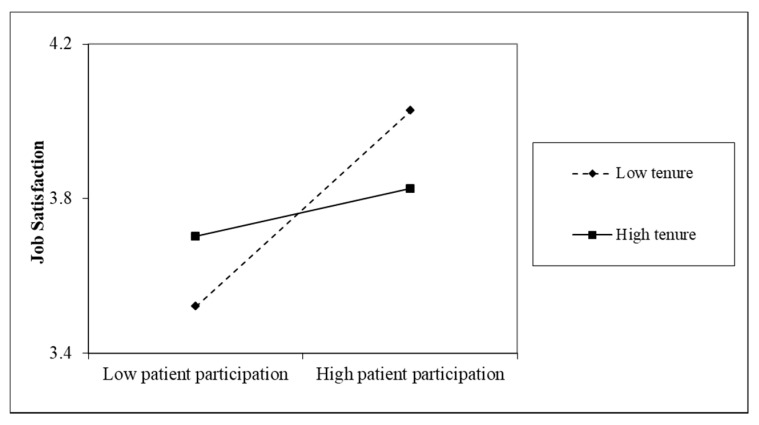
Simple slopes for the interaction between patient participation and tenure on nurse job satisfaction.

Hypothesis 4 proposed that nurses’ sociodemographic characteristics, including age, sex, education and organizational tenure, moderated the effect of patient participation on nurse job satisfaction, work engagement, and helping behaviors. Because our male nurse sample was too small (*n* = 7), we did not test sex as a moderator for the effect of patient participation on nurse outcomes. Model 2 in Table 5 shows that there was a significant interaction between patient participation and age on job satisfaction (*b* = −0.02, *p* < 0.05). To examine the simple effects, we used one standard deviation above and below the mean to denote high and low levels of age and patient participation, respectively. Figure 3 shows that for younger nurses, patient participation was significantly positively related to job satisfaction (*r* = 0.40, *p* < 0.001); for older nurses, patient participation was not related to job satisfaction (*r* = 0.08, *p* > 0.05). Model 3 in Table 5 shows that there was no interaction between nurse education level and patient participation on patient satisfaction. Model 4 in Table 5 shows that there was a significant interaction between patient participation and organizational tenure on job satisfaction (*b* = −0.00, *p* < 0.05). We probed the interaction and used one standard deviation above and below the mean to denote high and low levels of organizational tenure, respectively. Figure 4 shows that for nurses who had lower levels of organizational tenure, patient participation significantly increased their job satisfaction (*r* = 0.40, *p* < 0.001); for nurses who had higher levels of organizational tenure, patient participation was not significantly related to job satisfaction (*r* = 0.10, *p* > 0.05).

Table 6 and Table 7 show that there were no significant interactions between nurses’ sociodemographic characteristics and patient participation on work engagement and helping behaviors. Therefore, Hypothesis 4 was partially supported. 

## 5. Discussion

In this study, we investigated the effect of patient participation on patient satisfaction and nurse job satisfaction, work engagement, and helping behaviors, and its boundary conditions. Using data collected from 522 patients and 282 nurses from a public hospital in China, we found that for patients, the effect of patient participation on patient satisfaction depends on whether it is the first time of inpatient stay and the length of stay. For nurses, patient participation improves nurses’ job satisfaction, work engagement, and helping behaviors, and the effect of patient participation on job satisfaction is contingent upon nurses’ age and organizational tenure. Below, we discuss the theoretical and practical implications of our findings. 

### 5.1. Theoretical Contributions 

This research contributes to the literature by delineating a co-production dual process model of healthcare service between nurses and patients. Drawing upon the service-dominant logic perspective, we investigated service value co-creation between nurses and patients in the context of inpatient healthcare. First, both practice and academia stress the importance and necessity of involving patients in the healthcare service and treatment processes. Although a positive impact of patient participation on patient satisfaction has been widely found in various settings and with diverse samples, several studies have shown non-significant results [20,21,22]. This research endeavored to explain such non-significant findings by examining first inpatient stay and length of stay as two boundary conditions for effect of patient participation. We found that if it was the first hospitalization for patients, involving them in healthcare decreased their satisfaction; however, if it was not their first hospitalization, then there was no effect of patient participation on patient satisfaction. That is to say, first-time inpatients preferred not to participate in their treatment process—the more they were involved, the less satisfied or happy they were. We also found that patient participation only benefits those patients who stay in inpatient wards for a longer period, but not those who stay for a shorter period. A large number of studies have shown that patients prefer a passive role in healthcare service and treatment decision-making [6,35,36,37]. Our findings add insights to this point by showing that patient participation does not necessarily lead to favorable outcomes for first-time inpatients and patients with short hospitalization periods. 

Second, we demonstrated a positive effect of patient participation on nurses’ job satisfaction, work engagement, and helping behaviors. The results show that involving patients in healthcare and treatment not only benefits patients in certain situations, but it also rewards healthcare professionals. Working together with patients results in greater work enjoyment for nurses, they become more passionate and involved in their jobs, and they are willing to go out of the scope of their duties to help patients when needed. The communication between patients and nurses can be regarded as a channel for nurses to get feedback on their service. With patients’ input, nurses can gain a deeper understanding of how patients feel, what the patients expect, and where and how to improve their service. Ultimately, more desirable attitudes and behaviors of healthcare professionals are achieved. Our results offer more empirical evidence on the argument that the benefits of patient participation are universal for patients, healthcare professionals, and healthcare systems [1].

Third, examining the boundary conditions of patient participation on nurse outcomes reveals particularly interesting results. The effects of patient participation on work engagement and helping behaviors does not vary across different sociodemographic groups, suggesting that patient participation has a consistent impact on work engagement and helping behaviors among healthcare professionals. However, the effect of patient participation on job satisfaction is largely contingent upon nurses’ demographics, namely age and organizational tenure, and is particularly positive for younger nurses and those with shorter organizational tenure. This indicates that involving patients brings more benefits and value for these particular groups. For nurses of older age or with longer tenure, they are possibly more accustomed to the paternalist relationship between nurses and patients where patients engage in a rather passive role. Involving patients in the daily work somehow challenges their traditional way of dealing with patients, and therefore they may need some time to get used to more interaction between them and their patients. Younger nurses, however, are less influenced by the old-style patient–nurse relationship. Thus, they are more open to the changing role of patients and can better harness the benefits of involving patients in the process of healthcare delivery. Because our sample has a rather small portion of male nurses (*n* = 7), we did not test sex as a moderator for the effect of patient participation on nurse outcomes. Future research is encouraged to examine the moderating effect of gender with a larger sample of male nurses in order to have enough power.

Fourth, there are some other interesting findings in our results with regard to patient data. Our results revealed a negative effect of length of stay on patient satisfaction, indicating that the longer patients stay in the inpatient wards, the less satisfied they are. This finding echoes previous research, where patients with a longer length of stay are less satisfied with factors related to comfort, visiting, and cleanliness [38,39]. The possible explanation would be that patients who stayed longer are familiar with the healthcare delivery process, making them more critical or demanding about the service they receive. Another possible explanation is that severity of disease may affect both length of stay and patient satisfaction [12]. More sever disease makes hospitalization longer, and also potentially sabotages patients’ attitudes toward healthcare quality and overall satisfaction. However, in the current study, we do not measure the severity of disease, making our results correlational rather than causal. We also discuss this matter in the limitation section. Furthermore, we found that patients who stayed for the first time tended to be more satisfied with the healthcare service they received than those with prior hospitalization. The explanation would be that patients who have experienced prior hospitalization may have higher expectations for the healthcare service they receive. If their expectation is not met, they become disappointed and have a lower level of satisfaction. On the contrary, the expectation of first-time inpatients may not be that high, thus they are more likely to be satisfied. Severity of disease may also play a role here. Patients with more severe disease are more likely to have repeated admissions, and the low health status makes the patients depressed and less satisfied with the treatment they receive [19]. It is also noteworthy that our results revealed that high contact frequency between patients and nurses enhances patient satisfaction. Past research has shown that high contact frequency between nurses and patients is helpful for building good patient–nurse relationship and improving patient outcomes [40,41]. This research offers additional empirical evidence on the importance of contact frequency in healthcare.

The last point is related to the Chinese context. China now is undergoing a healthcare system reform aiming at delivering high quality and value-based healthcare service [3]. As argued in the Deepening Health Reform in China document published in 2016: “Patient empowerment and engagement is central to any health system reform that aims to improve efficiency and make providers accountable for the services they deliver.” ([3], p. 49). Although involving patients in healthcare has been reflected in a number of state policies in China, it will take a while to fully achieve it. This research, among the first few, provides empirical evidence on the benefits of patient engagement on patient satisfaction and healthcare professionals’ attitudes and behaviors. In this way, this research stresses the importance of engaging patients and citizens in healthcare, and furthermore, it lends confidence to the health care reform in China.

### 5.2. Practical Implications

This study offers important implications for management and policy implementation. First, in general, more policy, programs, and education should be initiated by associations, hospitals, and governments to further promote the notion of patient participation in healthcare, for example the “patient participation in hand hygiene program” and “patient safety program” from the World Health Organization (https://www.who.int/patientsafety/en/). In addition, the implementation of policy and practices should be at various levels, including the international level, national level, community level, organizational level, department level, and individual level so that the importance and benefits of patient participation are known to the public. 

Second, our results indicate that healthcare professionals should be conservative about involving first-time inpatients. Past research has shown that some patients prefer to be passive during the healthcare/treatment process. For example, they feel that active participation is not their role, and they merely want to get informed and receive information, preferring instead to give decision-making power to the healthcare professionals due to low health literacy [6,18,36]. In this case, it may not be appropriate to force patients to engage. Instead, healthcare professionals should put more effort into educating the patients and promoting the idea of patient participation. 

Third, our findings indicate that involving patients works better for young with shorter organizational tenure. Therefore, more managerial interventions should be placed on these groups to gain salient effects. For example, new employee training should emphasize the benefits of patient participation for new nurses, recent international and national policies on patient participation should be mentioned, current trends from both informal consultation and scientific enquiry should be covered, and so on. By doing so, nurses can better understand the priority of engaging patients. The goal is that they will then actively involve, educate, and engage their patients using effective communication. Ultimately, the healthcare systems and nurse–patient relationships would be improved.

### 5.3. Limitations and Future Research 

There are limitations of this research we would like to acknowledge. First, we collected our data from a single public hospital in China, so our results are more applicable for the healthcare system in public hospitals in the Chinese context. Future research could replicate and extend our findings in specialty hospitals, such as women’s hospitals and cancer hospitals. It would also be interesting to examine patient participation in private hospitals, or nursing home. 

Second, we collected both predictor and outcome variables from the same sources and all the variables were subjective. For example, when examining the effect of patient participation on nurse job satisfaction, work engagement and helping behaviors, all the data were provided by nurses and based on nurses’ subjective perceptions. Therefore, we are aware that this research involves the endogeneity problem. To address this problem, future research could use objective measures of patient and nurse outcomes, or use statistical remedy such as two-staged least squares to mitigate the problem of endogeneity. 

Third, due to pragmatic reasons, we were not able to match the dyadic patient–nurse relationship in the data collection. Future research could consider examining the effect of patient participation on patient and nurse outcomes, and connecting the outcomes of nurses and patients to establish a link. In doing so, a deeper understanding can be gained of what nurse attitudes or behaviors best predict patient satisfaction given the influence of patient participation. 

Fourth, past research suggests that the severity of disease affects the length of stay, readmission and patient satisfaction [12,20,42,43,44]. However, in the current study, we did not measure the severity of participants’ disease, rendering the results reported in this study correlational rather than causal. We encourage future efforts to include severity of illness as a predictor or a covariate when examining the effect of patient participation. 

Finally, the current study focused on the micro level, that is, the effect of patient participation on individual-level outcomes. Future research endeavors could be put on diverse levels, including macro, meso, and micro. It will be interesting to investigate patient participation at a higher level of analysis to enrich our knowledge of the impact of patient participation on groups, organizations, and even countries. 

## 6. Conclusions

Using service-dominant logic as the theoretical lens, this study investigates the co-production of healthcare service and service value co-creation between nurses and patients. We examine the effect of patient participation on patient satisfaction as well as nurse job satisfaction, work engagement, and helping behaviors. We further examine boundary conditions in these relationships. Using data from 282 nurses and 522 patients from a public hospital in China, we find that the effect of patient participation on patient satisfaction depends on prior hospitalization and length of stay. We also find that patient participation improves nurses’ job satisfaction, work engagement, and helping behaviors towards patients. Nurses’ sociodemographic characteristics moderate the effect of patient participation on job satisfaction, such that the effect is stronger for young nurses and those with shorter organizational tenure. We hope the current study fosters more research on patient participation.

## Figures and Tables

**Table 1:** Sociodemographic characteristics of the nurse sample.

Characteristics	Range/Category	Frequency	Percentage
Age	<25	127	44.56
	25–35	80	28.07
	36–45	19	6.65
	46–55	18	6.3
	>55	1	0.35
Sex	Female	275	97.52
	Male	7	2.48
Education	High school	12	4.26
	Technical degree	150	53.19
	Bachelor	111	39.36
	Master or above	9	3.19
Organizational tenure	<12 months	31	11.27
	12–36 months	69	25.08
	37–60 months	58	21.08
	61–120 months	56	20.33
	121–150 months	7	2.52
	151–240 months	38	13.81
	>240 months	17	6.16

Note: *n* = 282, due to missing values, sample size varies for each characteristic.

**Table 2:** Sociodemographic characteristics of the patient sample.

Characteristics	Range/Category	Frequency	Percentage
Age	<30	55	10.93
	31–40	85	16.87
	41–50	112	22.23
	51–60	95	18.85
	61–70	69	13.7
	71–80	58	11.52
	81–90	29	5.77
	>90	1	0.20
Sex	Female	279	53.45
	Male	243	46.55
Education	Below high school	140	27.29
	High school	133	25.93
	Technical degree	118	23.00
	Bachelor	97	18.91
	Master or above	24	4.68
First inpatient stay	No	342	68.95
	Yes	154	31.05
Length of stay	<3 days	111	24.94
	3–7 days	133	29.88
	8–14 days	98	22.02
	15–21 days	46	10.34
	22–28 days	25	5.62
	>28 days	32	7.19

Note: *n* = 522, due to missing values, sample size varies for each characteristic.

**Table 3:** Means, standard deviations, correlations, and internal consistency reliability.

Variables	Mean	S.D.	1	2	3	4	5	6	7	8	9
**Nurses**											
1. Job satisfaction	2.89	0.76	0.85								
2. Work engagement	3.31	0.68	0.41 ***	0.93							
3. Helping behaviors	3.68	0.73	0.23 ***	0.29 ***	0.87						
4. Patient participation	3.43	0.64	0.23 ***	0.28 ***	0.22 ***	0.82					
5. No. of patients contact	24.29	15.41	−0.07	0.02	−0.01	0.09	-				
6. Organizational tenure	83.82	79.10	−0.06	0.02	−0.14 **	−0.15 **	0.22 ***	-			
7. Age	29.35	8.35	−0.03	0.03	−0.14 **	−0.08	0.18 **	0.85 ***	-		
8. Sex	0.02	0.15	0.15 *	0.15 *	0.06	0.11 ^†^	−0.05	−0.07	−0.03	-	
9. Education	3.41	0.63	−0.00	0.02	−0.10	0.03	0.08	0.11 ^†^	0.07	−0.03	-
**Patients**											
1. Patient satisfaction	3.95	0.64	0.70								
2. Patient participation	3.53	0.42	0.12 **	0.93							
3. First inpatient stay	0.32	0.51	−0.05	−0.07	-						
4. Length of stay	9.52	8.45	0.06	−0.04	0.01	-					
5. Contact frequency	6.54	3.55	0.20 ***	−0.09 ^†^	0.09 ^†^	0.45 ***	-				
6. Age	52.24	16.87	−0.03	−0.11 *	−0.20 ***	0.13 *	0.08	-			
7. Sex	0.48	0.52	0.04	0.09 *	0.13 **	−0.03	0.02	0.03	-		
8. Education	2.47	1.21	0.10 *	0.16 ***	−0.08 ^†^	−0.08 ^†^	−0.11 *	−0.38 ***	0.07	-	

Note: Number of nurses = 282, number of patients = 522; Cronbach’s alphas are presented on the diagonal; ^†^ *p* < 0.10, * *p* < 0.05, ** *p* < 0.01, *** *p* < 0.001. S.D.: Standard deviation.

**Table 4:** Effect and boundary conditions of patient participation on patient satisfaction.

Variables	Model 1	Model 2
Patient participation	0.09 (0.10)	−0.05 (0.15)
First inpatient stay		2.37 (1.30) ^†^
Length of stay		−0.17 (0.07) *
Patient participation × First inpatient stay		−0.70 (0.38) ^†^
Patient participation × Length of stay		0.05 (0.02) *
Contact frequency	0.04 (0.01) ***	0.05 (0.01) *
Age	0.00 (0.00)	0.00 (0.00)
Sex	−0.01 (0.07)	−0.03 (0.07)
Below high school	−0.36 (0.19) ^†^	−0.45 (0.20)
High school	−0.24 (0.19)	−0.34 (0.20)
Technical degree	−0.20 (0.20)	−0.29 (0.20)
Bachelor	−0.14 (0.19)	−0.25 (0.20)
R square	0.06	0.09
F test	2.55 **	2.46 **

Note: *n* = 522; ^†^ *p* < 0.10, * *p* < 0.05, ** *p* < 0.01, *** *p* < 0.001.

**Table 5:** Interaction between patient participation and nurse sociodemographic on job satisfaction.

Variables	Model 1	Model 2	Model 3	Model 4
Patient participation	0.25 (0.07) ***	0.76 (0.24) **	0.26 (0.10) *	0.40 (0.10) ***
Age	0.00 (0.01)	0.07 (0.03) *	0.00 (0.01)	0.00 (0.01)
Sex	0.63 (0.29) *	0.61 (0.29) *	0.62 (0.29) *	0.60 (0.29) *
High school	−0.08 (0.34)	−0.05 (0.24)	0.32 (1.54)	−0.00 (0.10)
Technical degree	−0.01 (0.26)	−0.00 (0.10)	−0.12 (0.51)	−0.01 (0.26)
Bachelor	−0.01 (0.26)	−0.01 (0.26)	1.56 (1.59)	−0.83 (0.35)
Tenure	0.00 (0.00)	−0.00 (0.00)	0.00 (0.00)	0.01 (0.00) *
No. of patients contact	−0.01 (0.00) ^†^	−0.01 (0.00) *	−0.01 (0.00) ^†^	−0.01 (0.00) *
Patient participation × Age		−0.02 (0.01) *		
Patient participation × High school			−0.11 (0.43)	
Patient participation × Technical degree			0.03 (0.15)	
Patient participation × Bachelor			−0.43 (0.43)	
Patient participation × Tenure				−0.00 (0.00) *
R square	0.08	0.10	0.09	0.10
F test	2.86 **	3.13 **	2.17 *	3.06 ***

Note: *n* = 269; ^†^ *p* < 0.10, * *p* < 0.05, ** *p* < 0.01, *** *p* < 0.001.

**Table 6:** Interaction between patient participation and nurse sociodemographic on work engagement.

Variables	Model 1	Model 2	Model 3	Model 4
Patient participation	0.30 (0.06) ***	0.47 (0.21) *	0.30 (0.09) ***	0.37 (0.09) ***
Age	−0.00 (0.01)	0.02 (0.03)	−0.00 (0.01)	−0.00 (0.01)
Sex	0.58 (0.28) *	0.58 (0.28) *	0.58 (0.28) *	0.57 (0.27) *
High school	−0.16 (0.21)	−0.15 (0.21)	0.48 (1.37)	−0.14 (0.21)
Technical degree	−0.01 (0.21)	−0.01 (0.09)	−0.13 (0.46)	−0.01 (0.09)
Bachelor	−0.09 (0.23)	−0.08 (0.23)	0.70 (1.42)	−0.10 (0.23)
Tenure	0.00 (0.00)	0.00 (0.00)	0.00 (0.00)	0.01 (0.00)
No. of patients contact	−0.00 (0.00)	−0.00 (0.00)	−0.00 (0.00)	−0.00 (0.00)
Patient participation × Age		−0.01 (0.01)		
Patient participation × High school			−0.18 (0.39)	
Patient participation × Technical degree			0.04 (0.13)	
Patient participation × Bachelor			−0.22 (0.39)	
Patient participation × Tenure				−0.00 (0.00)
R square	0.10	0.10	0.10	0.11
F test	3.61 ***	3.29 ***	2.67 **	3.39 ***

Note: *n* = 268; *p* < 0.10, * *p* < 0.05, ** *p* < 0.01, *** *p* < 0.001.

**Table 7:** Interaction between patient participation and nurse sociodemographic on helping behaviors.

Variables	Model 1	Model 2	Model 3	Model 4
Patient participation	0.22 (0.09) ***	0.50 (0.23) *	0.26 (0.09) **	0.32 (0.09) ***
Age	−0.01 (0.01)	0.02 (0.03)	−0.01 (0.01)	−0.01 (0.01)
Sex	0.16 (0.27)	0.15 (0.22)	0.14 (0.28)	0.14 (0.27)
High school	0.52 (0.22) *	0.54 (0.23) *	1.15 (1.46)	0.54 (0.23) *
Technical degree	−0.03 (0.10)	−0.04 (0.10)	0.17 (0.49)	−0.03 (0.10)
Bachelor	−0.16 (0.24)	−0.17 (0.25)	1.45 (1.53)	−0.18 (0.25)
Tenure	−0.00 (0.00)	−0.00 (0.00)	−0.00 (0.00)	0.00 (0.00)
No. of patients contact	−0.00 (0.00)	−0.00 (0.00)	−0.00 (0.00)	−0.00 (0.00)
Patient participation × Age		−0.01 (0.01)		
Patient participation × High school			−0.18 (0.41)	
Patient participation × Technical degree			−0.06 (0.14)	
Patient participation × Bachelor			−0.44 (0.41)	
Patient participation × Tenure				−0.00 (0.00)
R square	0.09	0.10	0.09	0.10
F test	3.22 ***	3.01 ***	2.45 **	3.13 ***

Note: *n* = 269; *p* < 0.10, * *p* < 0.05, ** *p* < 0.01, *** *p* < 0.001.
